# Natural history of *Helicobacter pylori* VacA toxin in human gastric epithelium *in vivo*: vacuoles and beyond

**DOI:** 10.1038/s41598-017-15204-z

**Published:** 2017-11-06

**Authors:** Vittorio Necchi, Patrizia Sommi, Alessandro Vanoli, Roberto Fiocca, Vittorio Ricci, Enrico Solcia

**Affiliations:** 1 Department of Molecular Medicine, Pathologic Anatomy and Human Physiology Units, University of Pavia, Pavia, Italy; 2 Centro Grandi Strumenti, University of Pavia, Pavia, Italy; 3 Department of Surgical and Diagnostic Sciences, Pathology Unit, University of Genova and IRCCS S. Martino, Genova, Italy; 4 Pathologic Anatomy Service, Fondazione IRCCS Policlinico San Matteo, Pavia, Italy

## Abstract

Uptake, intracellular trafficking and pathologic effects of VacA toxin from *Helicobacter pylori* have been widely investigated *in vitro*. However, no systematic analysis investigated VacA intracellular distribution and fate in *H. pylori*-infected human gastric epithelium *in vivo*, using ultrastructural immunocytochemistry that combines precise toxin localization with analysis of the overall cell ultrastructure and intercompartimental/interorganellar relationships. By immunogold procedure, in this study we investigated gastric biopsies taken from dyspeptic patients to characterize the overall toxin’s journey inside human gastric epithelial cells *in vivo*. Endocytic pits were found to take up VacA at sites of bacterial adhesion, leading to a population of peripheral endosomes, which in deeper (juxtanuclear) cytoplasm enlarged and fused each other to form large VacA-containing vacuoles (VCVs). These directly opened into endoplasmic reticulum (ER) cisternae, which in turn enveloped mitochondria and contacted the Golgi apparatus. In all such organelles we found toxin molecules, often coupled with structural damage. These findings suggest direct toxin transfer from VCVs to other target organelles such as ER/Golgi and mitochondria. VacA-induced cytotoxic changes were associated with the appearance of auto(phago)lysosomes containing VacA, polyubiquitinated proteins, p62/SQSTM1 protein, cathepsin D, damaged mitochondria and bacterial remnants, thus leading to persistent cell accumulation of degradative products.

## Introduction

*Helicobacter pylori* is a Gram-negative bacterium that colonizes the stomach of about half the global population, thus being one of the most common bacterial infections worldwide^1–5^. *H. pylori*-dependent gastric pathology ranges from gastritis to mucosal gland atrophy or metaplasia and from peptic ulcer to neoplasia^3,4^. One of the most important virulence factors of this bacterium is a protein toxin named vacuolating toxin, VacA, because causing massive cytoplasmic vacuolation in cultured cells^1–3^.

An increasing body of evidence indicates that a functional crosstalk exists between VacA and another key virulence factor of *H. pylori*, the oncoprotein CagA (which is directly injected into host cells by the bacterium through a type IV secretion system) (reviewed in^2,6,7^). In some instances, these two virulence factors would antagonize each other (e.g., while CagA downregulates both vacuolating and proapoptotic effects induced by VacA, the toxin counteracts the effects of CagA on cell elongation and activation of the transcription factor NFAT and epidermal growth factor receptor). However, VacA and CagA would also act synergistically, for instance in providing *H. pylori* with specific nutrients (e.g., iron) required for its growth. This functional crosstalk would be finalized to achieve an optimal fitness of *H. pylori* with its ecological niche (i.e., the hostile gastric environment), limiting the overall cell damage caused and improving the infection efficiency of the bacterium^2,6,7^.

To intoxicate its main target host cells (i.e, gastric epithelial cells and T-lymphocytes) VacA exploits a peculiar intracellular trafficking pathway^2,5,8^. *In vitro* experimental data show that, after membrane binding, the toxin oligomerizes in lipid rafts and is then internalized through a clathrin-independent endocytic pathway devoted to internalization of glycosylphosphatidylinositol (GPI)-anchored proteins. VacA enters a tubulo-vesicular compartment, named GPI-anchored-protein-enriched early endosomal compartment (GEEC) or clathrin-independent carriers (CLICs), located close to the cell surface^2,9^. Delivered by a specific subset of early endosomes (motile because the formation of actin comet tails at their surface), most VacA then reaches late endosomes^5,10^. Here the pore-forming activity of VacA favors the accumulation of osmotically active ions (e.g., NH_4_^+^) followed by swelling. This leads to the namesake effect of VacA, the development of a massive cytoplasmic vacuolation^2,5^. In addition, actin-driven motility of VacA-containing endosomes favors toxin delivery to mitochondria, where VacA translocates and causes apoptosis^2,11^. However, the exact mechanisms through which VacA reaches its mitochondrial target is still largely unknown.

Kern *et al*.^12^ recently found that VacA reaches also the endoplasmic reticulum (ER) and the Golgi complex, thus identifying these organelles as novel target structures of the toxin. It remains however to be established how the toxin reaches ER and Golgi. Proteomic analysis of VacA-containing vacuoles (VCVs) purified from a T-cell line detected 122 VCV-specific proteins represented, in addition to typical endosomal/lysosomal proteins, by defined proteins from other organelles such as mitochondria, ER and Golgi^12^. The possibility thus arises that VCVs may exert a specific functional role in the intoxication process of the toxin, acting as a platform to trigger specific trafficking and signaling pathways exploited or influenced by VacA^8^.

VacA impairs immune responses, in particular by modulating the activity of immune effector cells like T lymphocytes, thus favoring the persistence of bacterial infection^13^. In addition, VacA has been found to inhibit antigen processing at endosomal level inside antigen-presenting cells (APCs)^14^. Cathepsin E has been identified as a crucial protease for antigen processing in APCs^15–17^ and has also been detected in gastric epithelium^18^ where its expression was markedly increased and expanded during *H. pylori* infection^19^. Thus, a possible VacA-sensitive role for cathepsin E in antigen processing inside gastric epithelial endosomes should be considered. Furthermore, VacA has been reported to specifically affect the host autophagic and lysosomal machinery, being also associated with autophagosomes, thus suggesting an additional role in the genesis of gastric epithelium pathologic changes^7,20,21^.

It must be underlined that most of the data reported derives from *in vitro* cellular models and that studies indicative of VacA interaction with, trafficking in, and action on the human stomach *in vivo* are still largely lacking. Thus, at least some of the results obtained so far should be interpreted with caution because, while they may make sense in terms of cell biology, it remains to be established whether, how and to what extent they have actual relevance in *H. pylori*-infected patients, especially considering the high cell, organ and species specificity of this bacterial infection.

The present study was specifically aimed to investigate the natural history of VacA in *H. pylori*-colonized human gastric epithelium *in vivo* by means of transmission electron microscopy (TEM) and ultrastructural immunocytochemistry, the optimal technology to investigate membrane-limited compartments like those involved in VacA intracellular trafficking and action^22,23^. In particular, we analyzed: a) *H. pylori* interaction with the gastric epithelium in delivering VacA toxin to host cells, b) VacA uptake at plasma membrane level, c) VacA intracellular trafficking and vacuole development, d) interorganellar interactions and presence of VacA in structurally normal or pathologically altered organelles other than endosomes/vacuoles, such as mitochondria, ER, Golgi, and cellular degradative structures like those of the autophagic-lysosomal pathway or the ubiquitin-proteasome system (UPS).

## Results

### VacA uptake and accumulation in endocytic-endosomal vesicles of H. pylori-infected human gastric epithelium

**Figure 1:**
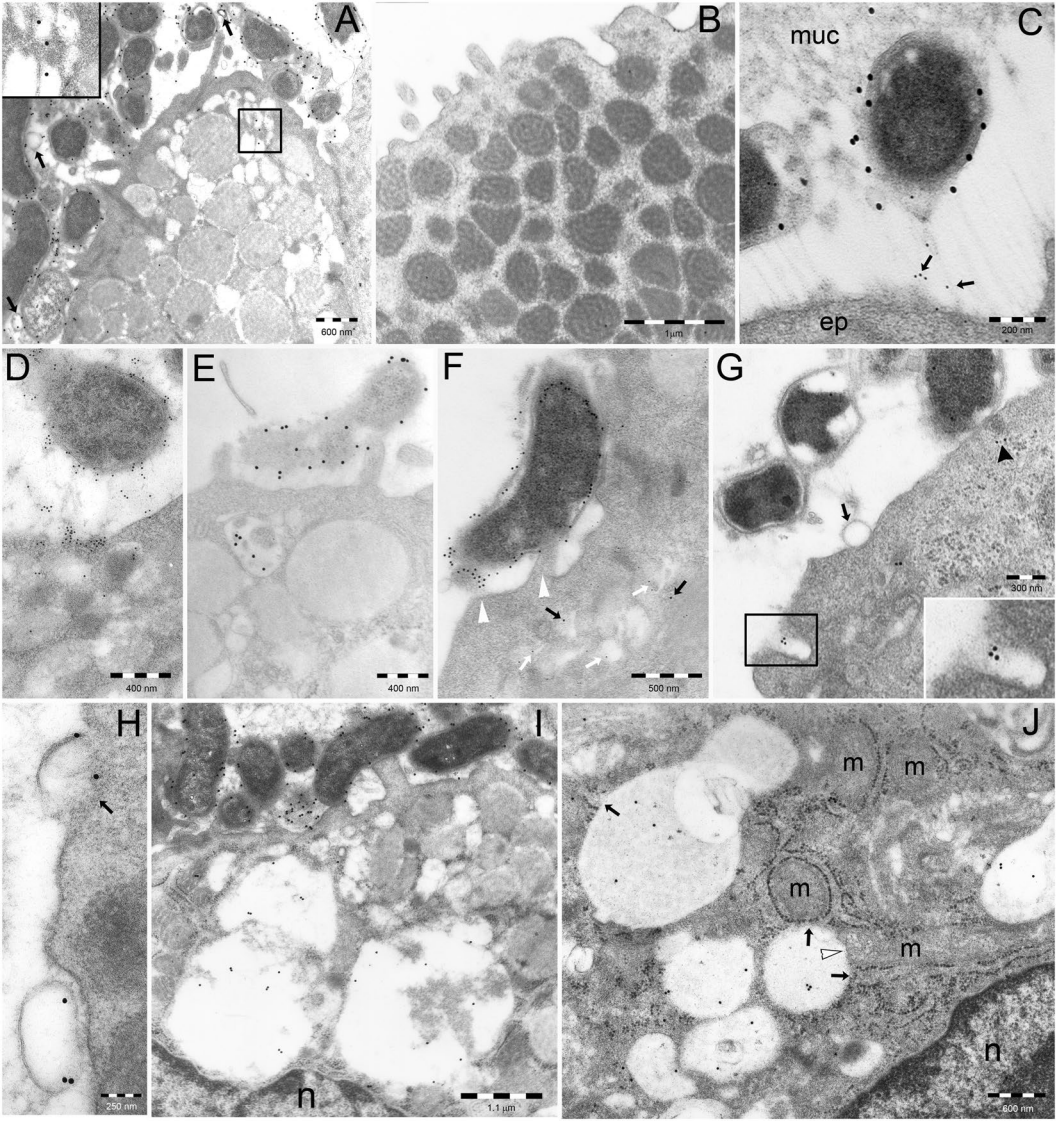
VacA uptake in endocytic-endosomal structures of *H. pylori*–infected human gastric epithelium. (**A**) VacA immunogold particles on bacterial outer membrane (see also **C**,**D**, and **E**) and a few OMVs (arrows). Inside the epithelium, note small clear vesicles with VacA reactivity (enlarged in the inset), subluminal or interspersed among mucin secretory granules. No such vesicles are seen in non-infected, VacA-negative, control epithelium (**B**). (**C**) An infected epithelium immunostained for VacA (20-nm gold particles) and *H. pylori* LPS O-antigen (10-nm particles) shows two *H. pylori* lying in the narrow space between luminal mucus (muc) and the epithelial surface (ep). In addition to VacA, mainly on bacterial outer membrane, LPS reactivity (arrows) is shown by filamentous material (fimbriae) connecting the external leaflet of bacterial outer membrane to the apical pole of epithelial plasma membrane. (**D**) VacA seems to slide along connecting filaments up to the epithelial membrane, where it is apparently taken up by immediately underlying endocytic vesicles. (**E**) Selective VacA uptake by a subluminal vesicle underlying an adhering bacterium. (**F**) VacA and LPS reactivities around a bacterium, which adheres to the epithelial membrane in its upper part and to two microvilli (arrowheads) forming two luminal pouches in between bacterial and epithelial plasma membrane. Both pouches and intraepithelial clear vesicles show VacA (black arrows) and/or LPS (white arrows). (**G**) An epithelial membrane invagination, enlarged in the inset to better recognize its VacA content and non-coated structure, exemplifies the endocytic process of toxin uptake. Note also VacA (arrowhead) in the cytoplasm underlying an adhering bacterium, and focal dilations of bacterial periplasmic space, a likely source of the OMV adhering to the epithelial membrane (arrow). (**H**) Two enlarged VacA-reactive OMVs interact with luminal epithelial membrane, either with (arrow) or without (lower vesicle) loss of bacterial outer membrane at site of adhesion. (**I**) Small subluminal endocytic/endosomal vesicles enlarge and fuse into VCVs in the deep supranuclear cytoplasm of an heavily infected cell. n, nucleus. (**J**) VCVs in the deep supranuclear cytoplasm open directly into ER cisternae (arrows), some of which envelop mitochondria (m). Direct VCV-mitochondria communication is also seen (white arrowhead). n, nucleus.

In infecting *H. pylori*, VacA immunoreactivity was mostly concentrated on bacterial outer membrane and its inner and outer leaflets (Fig. 1A,C–F). In addition, in agreement with previous findings on bacterial liquid culture^22^, VacA was found in some outer membrane vesicles (OMVs), ranging from 50 to 300 nm in size, detaching from bacteria and sometimes adhering to the epithelial membrane (Fig. 1A,G,H). Inside *H. pylori*-infected gastric epithelium, some clear vesicles filling subluminal cytoplasm and infiltrating in between mucin-filled secretory granules also showed VacA reactivity (Fig. 1A,D–F). VacA-reactive bacteria found in the same preparation served as positive controls for the reactivity of such clear vesicles (Fig. 1A,C,E). Neither such clear vesicles nor VacA reactivity were observed in the gastric epithelium from *H. pylori*-negative patients taken as a control (Fig. 1B). Bacterial adhesion to the epithelium was characterized either by direct contact, sometimes with apparent fusion, of the respective membranes (Fig. 1A,F), or by connecting thin fimbria-type filaments immunoreactive for LPS O-antigen and VacA (Fig. 1C,D). Both *H. pylori* products were seen to accumulate on the confronting luminal epithelial membrane (Fig. 1C,D) and to be taken up by subluminal clear vesicles (Fig. 1D,F).

**Figure 2:**
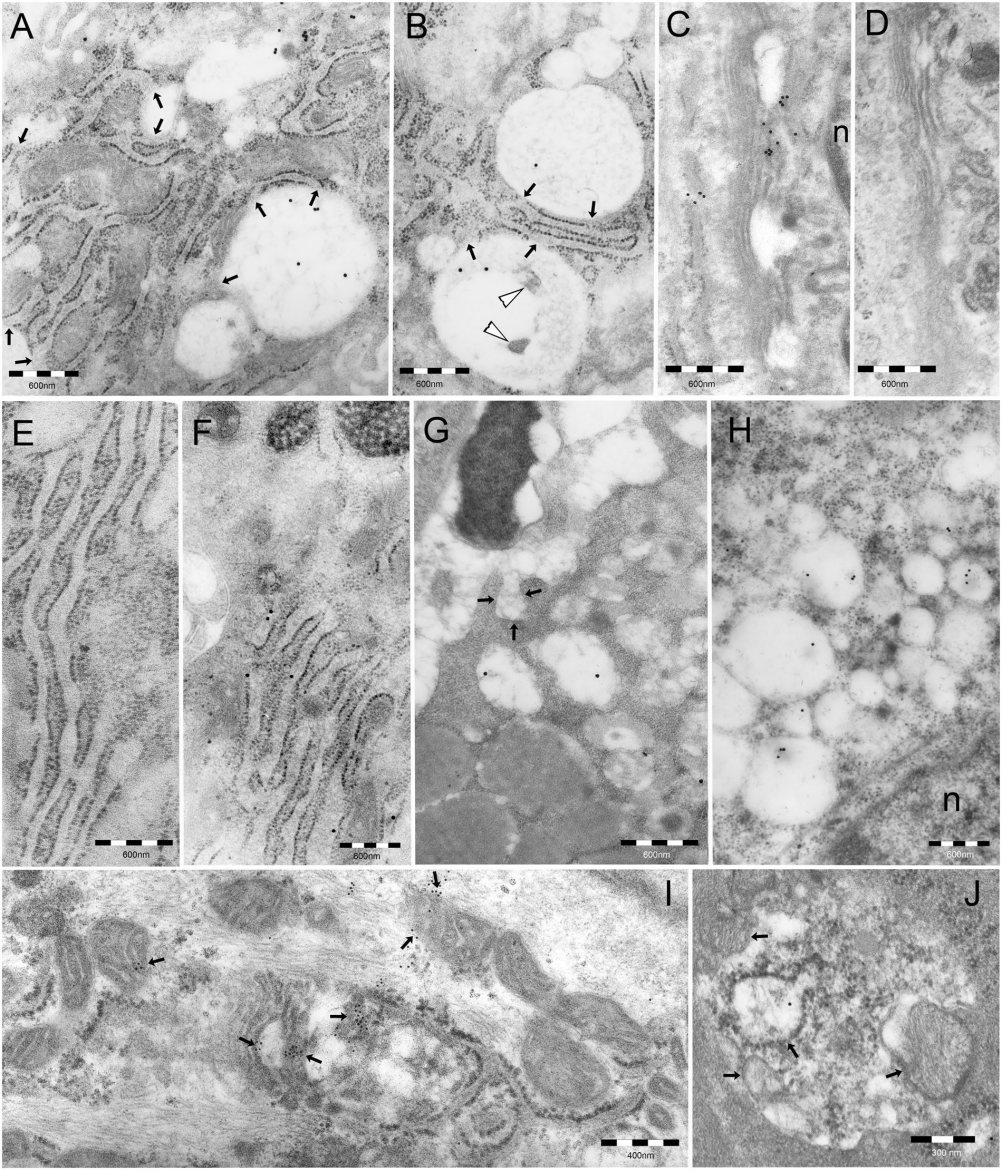
Intracellular distribution of VacA and damaged organelles. (**A**) and (**B**) VCVs imping on RER cisternae, several of which directly open into them (arrows). Note in (**A**) many well-preserved, VacA-unreactive mitochondria enveloped by RER cisternae and in (**B**) several endosomal vesicles fusing each other and with VCVs, in addition to lysosomal-type cellular debris (arrowheads) inside the lower VCV. (**C**) Juxtanuclear Golgi shows focal VacA reactivity and cisternae dilation. Also note VacA positivity of an adjacent ER cisterna. n, nucleus. Unreactive Golgi (**D**) and ER (**E**) are shown from uninfected control epithelium incubated with anti-VacA antibodies. (**F** to **H**) Cathepsin E reactivity, normally restricted to RER in *H. pylori*–negative epithelium (**F**, note unreactive secretory granules in the uppermost cytoplasm), extended to peripheral (**G**) and juxtanuclear (**H**) endosomes of a *H. pylori*–infected epithelium. n, nucleus. Note in (**G**) the unreactivity of the luminal bacterium and of a forming endocytic vesicle (arrows) for such a host protein. (**I**) Several mitochondria, partly enveloped by ER cisternae and focally VacA-reactive, are shown; a large one (center of the micrograph) is both VacA-positive (arrows) and heavily damaged (swelled and vacuolated). (**J**) Several heavily damaged mitochondria (arrows, one of which still VacA-positive) are enclosed in a cytoplasmic vesicle, likely of autophagosomal origin, followed by lysosomal fusion.

VacA-containing tubular invaginations of the luminal epithelial membrane of *H. pylori*-infected epithelium were found to penetrate cell cytoplasm and to interact with VacA-positive subluminal clear vesicles (Fig. 1F,G). These tubular invaginations were apparently lacking clathrin coat. They are likely to represent the human *in vivo* equivalents of the non-coated endocytic pits taking up VacA and generating VacA-positive superficial (early) endosomes (i.e., the aforementioned GEEC/CLICs) in experiments on cell lines *in vitro* ^2,9^. In addition, clear endosomal vesicles were frequently found to directly contact each other and with larger vacuoles, showing focal loss of their limiting membrane at contact site, a pattern highly suggestive for vesicle fusion (Figs 1A and 2A,B). This may provide a pathway for VacA trafficking to juxtanuclear (late) endosomes and their VCVs derivatives^2,10^.

### VCV role as VacA-distributing platform

VacA-accumulating VCVs were frequently found to directly contact ER, whose cisternae were also observed to open into VCVs, thus allowing free communication between respective lumina and possible transfer of VacA molecules from VCVs to ER (Figs 1J and 2A,B). As in turn ER cisternae frequently enveloped mitochondria (Figs 1J and 2A), this may well generate a pathway for VacA transfer also to mitochondria, in addition to the direct VCV-mitochondria communications we occasionally found (Fig. 1J). Of interest was also the finding of VacA reactivity in some Golgi cisternae and adjacent ER cisternae (Fig. 2C). In parallel tests, both the Golgi complex (Fig. 2D) and ER (Fig. 2E) of control gastric epithelium from uninfected human biopsies failed to show any VacA reactivity.

### Cathepsin E in endosomal vesicles and vacuoles

In normal, non-infected gastric epithelium, cathepsin E was found to be closely restricted to the rough ER (RER) (Fig. 2F). However, in *H. pylori*-infected gastric foveolar epithelium, cathepsin E was also detected in endosomes, both peripheral and juxtanuclear, and related VCVs (Fig. 2G,H).

### VacA, intracellular pathologic changes and cellular degradative structures

In addition to cytoplasmic vacuoles, several other pathologic changes were found in *H. pylori*-infected gastric epithelium, among which mitochondrial lesions, with loss of cristae or matrix lysis, and increased autophagy of damaged mitochondria (Fig. 2I,J). The close direct or ER-mediated interaction found between VCVs and mitochondria is likely to account for the focal VacA reactivity found in the latter organelles, which sometimes was directly coupled with pathologic changes (Fig. 2I,J).

**Figure 3:**
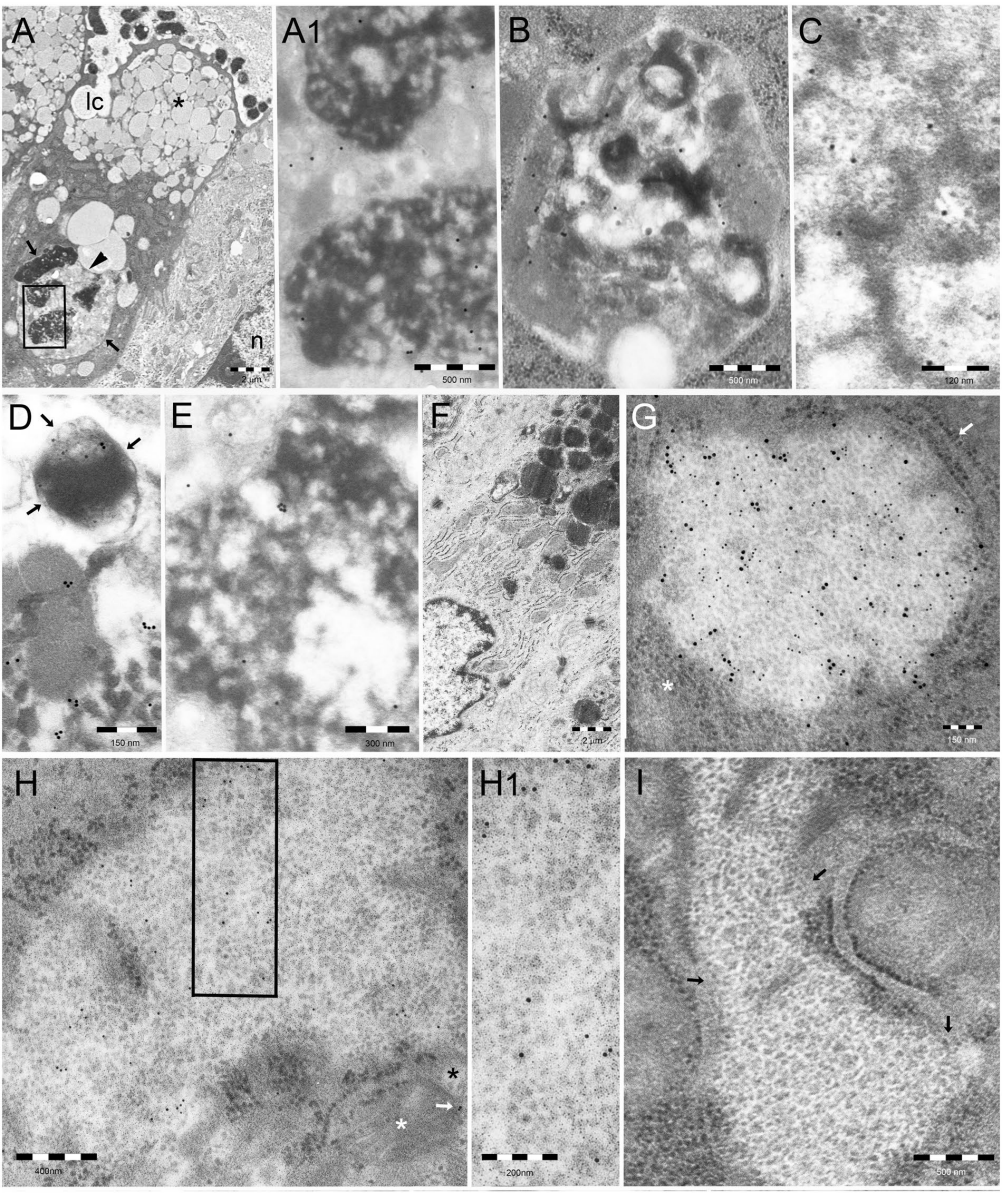
VacA-associated cellular degradative structures. (**A**) Well-developed, p62/SQSTM1 protein-reactive, ALIS-type auto(phago)lysosomal body (arrows) in the deep supranuclear cytoplasm of a *H. pylori*–infected cell also showing a penetrating luminal cleft (lc), a subluminal collection of mucin granules (asterisk) with interspersed small clear endosomal vesicles and deep VCVs directly interacting (arrowhead) with such a body. This is partly (see box in A) enlarged in (A1) to show p62/SQSTM1-reactivity and heterogeneous lysosomal-type ultrastructure. n, nucleus of a cell adjacent to the body-bearing one. (**B**) Selective immunoreactivity for LC3 protein of another ALIS-type body further supports its auto(phago)lysosomal nature. (**C** to **E**) High enlargements of part of three auto(phago)lysosomal bodies to show their cathepsin D (**C**), VacA (**D**) and K63-linked pUb chains (**E**) reactivity. Also note in (**D**) the presence inside the body of a VacA-reactive phagocytosed *H. pylori* (arrows). (**F**) Neither auto(phago)lysosomes nor VCVs were found in the supranuclear cytoplasm (mainly filled by VacA-unreactive RER) of control, uninfected gastric epithelium. (**G**) A PaCS, formed by a collection of barrel-like, proteasome-reactive particles (see positivity for 19 S proteasome subunit by 10-nm immunogold), is found in the cytoplasm below the nucleus of a *H. pylori*–infected foveolar cell, surrounded by free ribosomes (asterisk) and RER cisternae (arrow). Also note in (**G**) selective PaCS reactivity for the polyubiquitinated protein-specific FK1 antibody (20-nm gold). (**H**) Focal PaCS reactivity for VacA (enlarged in H1). Note VacA reactivity (white arrow) also at the level of a mitochondrion (white asterisk) enveloped by an ER cisterna (black asterisk). (**I**)The unreactivity of PaCS for K63-linked pUb chains is shown; also note direct merging of several RER cisternae with the PaCS (arrows).

A frequent finding, especially in cells with consistent cytotoxic changes (larger endosomal vacuoles, damaged mitochondria, autophagic vesicles, lysis of mucin granules or penetrating intercellular clefts), were supranuclear bodies (Fig. 3A) of vesicular to solid structure, often containing dense cathepsin D-positive lysosome-type deposits. Such single-membrane-limited bodies, which occasionally stored bacterial or mitochondrial remnants, showed reactivity for p62/SQSTM1 protein, LC3, VacA, cathepsin D, LPS, and K63-linked polyubiquitin (pUb) chains (Fig. 3A–E). Thus, they were interpreted as auto(phago)lysosomes. Notably, no such bodies were found in control uninfected epithelium (Fig. 3F).

Particle-rich cytoplasmic structures (PaCSs)^24^, characterized by a collection of barrel-like particles heavily reactive for 19 S and 20 S proteasome, were detected in the cytoplasm below the nucleus (Fig. 3G), especially in cells showing less prominent cytotoxic changes and scarce or no auto(phago)lysosomes. Such structures were also intensely positive for FK1 antibody-reactive polyubiquitinated proteins (Fig. 3G) and, moderately/focally, for VacA (Fig. 3H), while they were unreactive for K63-linked pUb chains (Fig. 3I) and p62/SQSTM1 protein (not shown). They thus reproduced structural and cytochemical patterns of the PaCSs previously seen in *H. pylori* gastritis, gastric cancer and several cell lines^24–26^, including their close topographic relationship with surrounding ribosomes and RER cisternae (Fig. 3G–I). Unfortunately, despite testing different antibodies raised against the K48-linked pUb chains (i.e., the type of pUb chains known to be selectively associated with proteasomal degradation^27^, we did not find any antibody which worked in our TEM experimental conditions (i.e., aldehyde-osmium fixed resin-embedded specimens). This prevented a direct proof of K48-linked pUb nature of the FK1-positive polyubiquitinated proteins stored by PaCSs.

## Discussion

A prominent finding of this investigation of *H. pylori*-infected human gastric epithelium *in vivo* was the detection of a population of small subluminal vesicles (and/or tubulovesicles), interposed with or overlying mucin granules, which were essentially lacking in non-infected epithelium. The actual presence of VacA immunoreactivity in a substantial fraction of such vesicles as well as in endocytic pits of the luminal plasma membrane strongly supports the endocytic-endosomal nature of such vesicles and their likely induction by bacterial infection, as previously suggested by *in vitro* experiments^22,28–30^. The non-coated tubular nature of these endocytic pits suggests that the clathrin-independent non-caveolar pinocytic mechanism^31^ of VacA internalization documented in experimental models *in vitro* ^9,32^ may have an *in vivo* counterpart in *H. pylori*-infected patients. Given their abundance in the infected epithelium, especially in association with *H. pylori* intimately adhering to surface epithelial cells, it seems likely that the endocytic-endosomal vesicles represent the main route of VacA cellular uptake *in vivo*. Although VacA-containing OMVs as well as whole *H. pylori* bodies were also found to intimately adhere with and enter epithelial cell lines *in vitro* and the gastric epithelium *in vivo* ^22,23,33,34^ (the present study), these remained less common findings than VacA-containing endocytic-endosomal vesicles inside the cells of *H. pylori*-infected gastric biopsy samples here studied. Our immunocytochemical findings also suggest that endosomal vesicles represent the main route of VacA intracellular trafficking, from subluminal early to juxtanuclear late endosomes, where most VCVs accumulate *in vivo*.

Of special interest is our observation of VCVs frequently contacting and also opening into adjacent ER cisternae. Of high interest is also the observation of close simultaneous interactions of VCVs with ER cisternae and mitochondria, of which here we provide the first *in vivo* demonstration as VacA targets. Our findings thus provide the *in vivo* counterpart in *H. pylori*-infected human epithelium of the *in vitro* observations by Kern *et al*.^12^ in non-gastric cell lines (i.e., Jurkat T-cell and epithelial HeLa cell lines) showing that VacA also targets ER and Golgi and suggesting that VCVs may have a key role in VacA intoxication processes beyond the vacuoles. Indeed, VCVs seem to act as a platform to trigger specific trafficking pathways exploited by the toxin. Our direct immunocytochemical detection of VacA inside Golgi cisternae further confirms Kern *et al*.’s data^12^, although the exact route of VacA transport to the Golgi (from ER to Golgi?) remains unknown. Our *in vivo* findings also support Calore *et al*.’s *in vitro* observations^35^ suggesting that VacA might be transferred to mitochondria by endosomal–mitochondrial juxtapositional exchange.

Intriguingly, the existence of distinct interaction domains between the ER and other organelles (such as mitochondria and endosomes), known as membrane contact sites (MCSs), has been recently demonstrated (reviewed in^36^). At MCSs, organelle membranes are closely apposed and tethered (but apparently do not fuse), and here various protein complexes might work in concert to perform specialized functions as binding, sensing and transferring molecules, as well as engaging in organelle biogenesis and dynamics^36^. Through the establishment of such physical contacts with different cell organelles, ER seems thus emerging as a key player in spaziotemporal control of organellar dynamics inside the cell. It has been speculated that these interorganellar “synapses” might serve as a direct delivery route between compartments, bypassing usual trafficking pathways known so far^37^. Whether canonical MCSs may have a role in VacA trafficking it remains to be investigated.

The endosomal accumulation of VacA toxin and other *H. pylori* antigens, like LPS O-antigen, is especially interesting considering 1) the well-known role of endosomes in taking up, storing and processing antigens to be membrane-presented in HLA molecules background^38,39^, 2) the high *de novo* expression of HLA-DR by *H. pylori*-infected gastric epithelium^19^, 3) the capacity of VacA to interfere with antigen processing by professional APCs at endosomal level^14^, as well as 4) our present finding of endosomal localization of cathepsin E, an aspartic protease crucial for antigen processing in several APCs^15–17,40,41^. A VacA-sensitive role for cathepsin E in *H. pylori* antigen processing at endosomal level would account for the expression increase and expansion (including endosomal involvement in addition to RER) of this protease we found in the infected gastric epithelium^19^ (this study). This would also suggest the VacA- and cathepsin E-storing endosomal compartment as an appropriate site for the inhibitory action of VacA on cell processing of *H. pylori* antigens.

Several pathologic changes have been reported in cell lines incubated with *H. pylori* or its VacA toxin including, besides VCVs^28–30,42,43^, mitochondrial lesions^11,44,45^, and altered autophagic/lysosomal processes^7,20,21^. We found that all such changes occurred also *in vivo* in the infected human gastric epithelium, although cell vacuolation was less prominent *in vivo* than that observed in non-confluent cell monolayers *in vitro*, while being more akin to that seen in confluent cell monolayers^46^ (our unpublished data). In addition, we provided direct immunocytochemical evidence for *in vivo* accumulation of VacA toxin inside swelled endosomes (i.e., VCVs) as well as mitochondria, which in turn also showed structural damage. The latter finding seems especially relevant as VacA has been shown to damage mitochondria *in vitro* causing mitochondrial membrane depolarization and cytochrome *c* release^11,45^. This in turn may potentially trigger mitophagy, the selective form of autophagy targeting damaged mitochondria to limit cell damage and prevent cell death^7,47^. In this respect, here we provide direct *in vivo* evidence of autophagic degradation of damaged mitochondria containing VacA.

VacA activity was found to persist for a long time inside cultured gastric epithelial cells^48^ whose vacuoles showed ultrastructural and cytochemical patterns of both late endosomes and lysosomes^28,29,49^ and, especially after prolonged incubation time (i.e., 24 hours or more), also showed autolysosomal features^28,50^. These *in vitro* findings well fit with present *in vivo* detection of close topographical and cytochemical correlations between VCVs and supranuclear auto(phago)lysosomal bodies.

Aggresome-like induced structure (ALIS)-type bodies reactive for polyubiquitinated proteins and p62/SQSTM1^51^ have been reported by confocal microscopy in gastric epithelium of mice infected with a mouse-adapted *H. pylori* strain^52^. Apparently similar pUb-storing bodies have been characterized ultrastructurally as autophagosomes/autolysosomes in LPS-matured dendritic cells (DCs)^53,54^ and macrophages^55^, and suggested to be storage site of potentially antigenic polyubiquitinated proteins to be processed and membrane-presented by such cells^54,56,57^. In the present study, we ascertained at ultrastructural level the development in *H. pylori*-infected human gastric epithelium of cytoplasmic bodies reactive for K63-linked pUb chains, p62/SQSTM1 protein, and LC3 protein and obtained direct evidence of a *H. pylori*-related origin of such bodies by detecting bacterial remnants, toxins, and antigens inside them. The presence of vesicular membranes, LC3 and p62/SQSTM1 proteins, and K63-linked pUb chains is highly suggestive for an autophagic component^53,58–60^. Furthermore, we also obtained ultrastructural and cytochemical (e.g., cathepsin D reactivity) evidence for a lysosomal contribution to their genesis. Taken together, our findings support an auto(phago)lysosomal nature of VacA-containing ALIS-type bodies developing in *H. pylori*-infected human gastric epithelium. A role for VacA itself in the origin and persistence of auto(phago)lysosomal bodies may be considered, given the toxin-induced loss of cathepsin D activity, crucial for lysosomal function^21,61^. Our detection of *H. pylori* LPS in ALIS-type auto(phago)lysosomal bodies is also worth noting, given the role played by bacterial LPS in their genesis in human DCs^54^.

Supranuclear auto(phago)lysosomal bodies were more frequently found in cells showing prominent cytotoxic lesions, while being scarce or absent in those lacking such changes. This supports a role of VacA-dependent cytotoxicity, with special reference to damaged organelles, in the genesis of auto(phago)lysosomes, in keeping with previous observations *in vitro* concerning the fate of VacA-induced vacuoles^28,50^.

The UPS-rich PaCS is a focal collection of proteasome barrel-like particles, polyubiquitinated proteins and heat-shock proteins identified in some neoplastic or fetal tissues and cell lines^24–26,54,55^. This structure, specifically arising inside ribosome-rich cytoplasm below the nucleus, is likely to have a role in quality control and degradation of misfolded, mutated or anyway damaged cytosolic proteins, known to be produced in excess in neoplastic or fetal cells as well as in hematopoietic cells specifically stimulated by trophic factors and interleukins^25,26,54^. Our detection of PaCSs in *H. pylori*-infected epithelium might be suggestive of an altered protein turnover resulting in UPS stress.

In *H. pylori*-infected human gastric epithelium, unlike auto(phago)lysosomal bodies, PaCSs were usually found in the cytoplasm below the nucleus and, preferentially, in cells showing limited cytotoxic changes. Concerning the polyubiquitinated proteins they store, PaCSs differed from autophagosomes/autolysosomes in being unreactive for K63-linked pUb-directed antibodies, while being reactive for the FK1 antibody, known to recognize *in vitro* polyubiquitinated proteins exhibiting either K63- or K48-linked pUb chains^62^. As only K48-linked polyubiquitinated proteins are known to selectively associate with proteasome^27^, it seems likely that the FK1-reactive and K63-linkage-unreactive polyubiquitinated proteins associated with proteasome particles inside PaCSs are to be interpreted as K48-linked proteins. Interestingly, the direct opening we found of some ER cisternae into PaCSs may indicate a pathway through which VacA reaches this essentially cytosolic UPS-rich structure and, more in general, might also suggest a way for endocytosed exogenous antigens to reach cytosolic proteasome for class I cross-presentation^54,63^.

Our present findings show that, notwithstanding their common association with *H. pylori* infection and common storage of polyubiquitinated proteins, ALIS-type auto(phago)lysosomal bodies and PaCSs are cytochemically linked to two different protein-degradative pathways, namely: 1) the K63-linked pUb, p62/SQSTM1 and LC3 positive autophagic-endolysosomal system, and 2) the proteasome and K48-linked pUb chain positive UPS, respectively. In keeping with our recent findings in human DCs *in vitro* ^54^, PaCSs may represent an early, chaperon protein-promoted and ubiquitin/proteasome-mediated cellular attempt to repair or degrade *H. pylori*-induced misfolded proteins, whereas the ALIS bodies may result from cellular activation of the autophagic/lysosomal pathway by severe cytotoxic lesions affecting cytoplasmic organelles.

In conclusion, our *in vivo* study shows that VacA mainly enters *H. pylori*-infected human gastric epithelium by endocytosis and accumulates into endosomes and endosome-derived VCVs, which directly communicate with ER cisternae and ER-enveloped mitochondria. This latter finding supports toxin trafficking from VCVs to other organelles such as ER/Golgi and mitochondria, as previously suggested by *in vitro* experiments. *De novo* endosomal expression by infected gastric epithelium of the antigen-processing proteinase cathepsin E may have a role in the complex VacA-associated host immune-inflammatory response which characterizes *H. pylori* infection. VacA-induced cytotoxic effects on cell organelles and protein turnover is associated with activation of main cellular degradative systems with persistent accumulation of degradative products inside auto(phago)lysosomes.

## Materials and Methods

### Human biopsy samples

We reinvestigated biopsy samples of gastric mucosa taken in the period 1981–95 from 26 patients (15 males and 11 females, aged between 26 and 79 years) undergoing routine endoscopic and histologic examination for dyspepsia as requested by the physician in charge of the patient and with the written consent of the patient^24^. The study has been approved by the Ethics Committee of Fondazione IRCCS Policlinico San Matteo (Pavia, Italy) as a reinvestigation of archival material along the same line (i.e., diagnosis of *H. pylori*-dependent gastritis) as for the original written consensus. All the methods were performed in accordance with the relevant guidelines and regulations.

Six biopsy specimens (3 from the antrum and 3 from the corpus of the stomach) were taken from each patient. From each biopsy site, 2 samples were processed for light microscopy and 1 sample for TEM. Fifteen patients resulted *H. pylori*-positive in all biopsies at both light microscopy (Giemsa staining and histochemistry for *H. pylori* LPS) and TEM (detection of characteristic bacterial ultrastructure coupled with VacA and *H. pylori* LPS cytochemistry) investigation. Their biopsy specimens were thus judged as suitable for present investigation on VacA interaction with human gastric mucosa. Four patients resulted *H. pylori*-negative in all biopsies from the antrum and corpus, extensively investigated at both light microscopy and TEM as above. The eight TEM-processed biopsy specimens from these 4 patients were thus taken as negative controls in the present study. Biopsy specimens from the remaining 7 patients showed more limited *H. pylori* colonization, often unequally distributed among different specimens. These cases were not further investigated in the present study.

### TEM and ultrastructural immunocytochemistry

For TEM investigation, biopsy samples were fixed for 4 hours with 2% formaldehyde and 2.5% glutaraldehyde in 0.1 M phosphate buffer (pH 7.3), followed by 1% osmium tetroxide for 1 hour, and then embedded in Epon-Araldite resin^24^. Thin (~70 nm) sections were stained with uranyl-lead or underwent the immunogold procedure followed by uranyl-lead counterstaining, as previously detailed^22,23,64^. Specimens were analyzed by a Jeol JEM-1200 EX II transmission electron microscope equipped with an Olympus CCD camera (Mega View III). Images were processed and assembled by using the Adobe Photoshop CS5 software.

### Antibodies

The following primary antibodies (Abs) were used: 1) Anti-VacA: a) rabbit polyclonal 123 antiserum raised against purified VacA (s1/i1/m1 *vacA* genotype)^65^ (kindly provided by T.L. Cover, Nashville, TN) and b) rabbit polyclonal HPP-5013-9 Ab (Austral Biologicals, San Ramon, CA) raised against a recombinant VacA fragment (aa 311-819); 2) Anti-LPS O-antigen: rabbit polyclonal V4074 Ab (Biømeda, Foster City, CA) (Andersen 1988 APMIS); 3) Anti-cathepsin E: a) rabbit polyclonal PA3-16821 Ab (Thermo Fisher Scientific, Rockford, IL), and b) mouse monoclonal sc-166343 Ab (clone E-8; Santa Cruz Biotechnology, Santa Cruz, CA); 4) Anti-cathepsin D: a) mouse monoclonal NPB1-04278 (clone 4G2; Novus Biologicals, Littleton, CO), and b) rabbit polyclonal sc-10725 Ab (Santa Cruz Biotechnology); 5) Anti-p62/SQSTM1: a) rabbit polyclonal PM045 Ab (MBL, Nagoya, Japan), and b) mouse monoclonal sc-28359 (clone D-3; Santa Cruz Biotechnology); 6) Anti-LC3: rabbit polyclonal NB100-2220 Ab (Novus Biologicals); 7) Anti-polyubiquitinated proteins: mouse monoclonal FK1 Ab (BML-PW8805; Enzo Life Sciences, Farmingdale, NY); 8) Anti-K63-linked pUb chains: mouse monoclonal BML-PW0600 Ab (clone HWA4C4; Enzo Life Sciences); 9) Anti-K48-linked pUb chains: a) rabbit monoclonal 05-1307 Ab (clone Apu2; Millipore, Billerica, MA), b) rabbit monoclonal 140601 Ab (clone EP8589; Abcam, Cambridge, UK); c) rabbit monoclonal A-101 Ab (clone 1001c; BostonBiochem, Cambridge, MA); 10) Anti-20S proteasome α/β subunits: rabbit polyclonal BML-PW8155 (Enzo Life Sciences); 11) Anti-19S proteasome S2 subunit: rabbit polyclonal 539166 Ab (Calbiochem, La Jolla, CA).

As secondary Abs, anti-rabbit or anti-mouse immunoglobulins labeled with 10, 15 or 20 nm gold particles (British Bio Cell, Cardiff, UK, and Aurion, Wageningen, The Netherlands) were used^23,24,26^.

Tests to evaluate the specificity of immunogold labeling were carried out using antibodies absorbed with excess antigen and omitting or substituting the specific antibodies in the first layer of the immunogold procedure. Positive and negative controls were obtained by parallel investigation of *H. pylori* cultures, epithelial cell cultures, and *H. pylori*-positive or -negative gastric mucosa specimens as in previous studies^22,23^. In particular, both anti-VacA antibodies used were tested using parallel TEM investigation on well-characterized bacterial cultures either VacA-producing (*H. pylori* strains 60190, ATCC 49503, and CCUG 17874, from Culture Collection University of Göteborg, Sweden) or not producing the toxin (*H. pylori* strain 60190:v1, the isogenic mutant of the 60190 strain in which the *vacA* gene was disrupted by insertional mutagenesis, kindly provided by T.L. Cover, Nashville, TN). We also assessed the specificity of these antibodies by means of SDS-PAGE, followed by Western blotting, on bacterial lysates and broth culture filtrates of the aforementioned *H. pylori* strains.

### Data availability

No datasets were generated or analysed during the current study.
